# Backbone chemical shift and secondary structure assignments for mouse siderocalin

**DOI:** 10.1007/s12104-024-10171-9

**Published:** 2024-04-02

**Authors:** Johanna Moeller, Nina G. Bozhanova, Markus Voehler, Jens Meiler, Clara T. Schoeder

**Affiliations:** 1 Institute for Drug Discovery, Leipzig University Medical School, 04103 Leipzig, Germany; 2 Center for Scalable Data Analytics and Artificial Intelligence (ScaDS.AI) Dresden/Leipzig, Leipzig University, Leipzig, Germany; 3 Center for Structural Biology, Vanderbilt University, Nashville, TN 37232 USA; 4 Department of Chemistry, Vanderbilt University, Nashville, TN 37232 USA

**Keywords:** *Mus musculus*, Siderocalin, Lipocalin 2, Secondary structure prediction

## Abstract

The lipocalin protein family is a structurally conserved group of proteins with a variety of biological functions defined by their ability to bind small molecule ligands and interact with partner proteins. One member of this family is siderocalin, a protein found in mammals. Its role is discussed in inflammatory processes, iron trafficking, protection against bacterial infections and oxidative stress, cell migration, induction of apoptosis, and cancer. Though it seems to be involved in numerous essential pathways, the exact mechanisms are often not fully understood. The NMR backbone assignments for the human siderocalin and its rat ortholog have been published before. In this work we describe the backbone NMR assignments of siderocalin for another important model organism, the mouse - data that might become important for structure-based drug discovery. Secondary structure elements were predicted based on the assigned backbone chemical shifts using TALOS-N and CSI 3.0, revealing a high content of beta strands and one prominent alpha helical region. Our findings correlate well with the known crystal structure and the overall conserved fold of the lipocalin family.

**Supplementary Information:**

The online version contains supplementary material available at 10.1007/s12104-024-10171-9.

## Biological context

Siderocalin, also known as lipocalin-2, NGAL or 24p3, is a glycoprotein from the lipocalin family. Members of this family are defined by a conserved overall structure but show diverse functionalities and no significant sequence conservation (Flower et al. 2000). The structure consists of an anti-parallel β barrel forming a cavity that is ideal for high-affinity small molecule binding as well as a conserved α helix (Flower 1996). In contrast to the majority of proteins in the lipocalin family that show a preference for hydrophobic ligands due to the apolar lining of the binding site, the calyx of siderocalin is lined with polar and positively charged amino acids, resulting in increased affinities to polar, highly substituted catecholate-type ligands (Goetz et al. 2002). Siderocalin is involved in various biological processes like cellular immunity (La Manna et al. 2014), inflammation (Abella et al. 2015), metabolic homeostasis and insulin resistance (Guo et al. 2010). It has also been reported to play a role in the regulation of oxidative stress as well as in iron trafficking (Xiao et al. 2017). While siderocalin cannot bind iron directly, it can interact with siderophores, small iron-chelating molecules produced by bacteria and fungi (Neilands 1995). Iron is an essential micronutrient for many of these microorganisms, and siderophores enable them to scavenge it from their surroundings. By binding the iron-chelating siderophores, like enterobactin from *E. coli*, siderocalin is executing its antibacterial activity (Goetz et al. 2002).

Siderocalin orthologs in human (also called human neutrophil gelatinase-associated lipocalin, human neutrophil lipocalin), rat (*neu*-related lipocalin, α_2_-microglobulin-related protein), and mouse (24p3, 24 kDa superinducible protein, uterocalin) are of special interest because of their clinical relevance and their presence in established model organisms (Goetz et al. 2002). Sequence identity of human to rat and mouse siderocalin is ca. 60%, whereas mouse and rat share a sequence identity of 81% (Åkerström 2006). Despite the difference in sequences, the experimental structures of human, rat, and mouse siderocalins (Bandaranayake et al. 2011; Goetz et al. 2000; Zhang et al. 2009) demonstrate not only the conserved fold of the lipocalin family but show also a high structural similarity with each other. The Cα root-mean-square deviation (RMSD) between human and mouse siderocalin is 1.01 Å, human and rat − 2.03 Å, mouse and rat − 1.95 Å, respectively, as determined with the matchmaker tool in ChimeraX (Meng et al. 2006).

The NMR backbone assignment is already available for the human (Coles et al. 1999) and rat orthologs (Zhang et al. 2009). Here we present the near complete backbone assignment for mouse siderocalin.

## Methods and experiments

### Protein expression and purification

The mouse siderocalin (mScn) DNA sequence (Uniprot accession number P11672) fused to a His-tagged B1 domain of Streptococcal protein G (GB1) and codon-optimized for *Escherichia coli* was cloned into pET21a plasmid. The plasmid was transformed into *E. coli* BL21(DE3). A transformed colony was grown in 55 mL LB medium containing natural abundance isotopes with 0.1 g/L ampicillin until OD_600_ reached 0.8. 50 mL of the cell suspension were centrifuged and the cell pellet was transferred into 1 L M9 minimal medium containing 1 g/L ^15^NH_4_Cl and 2 g/L ^13^C glucose as the sole nitrogen and carbon sources as well as 0.1 g/L ampicillin. At OD_600_ 0.8 protein expression was induced with 0.2 mM IPTG, and cells were harvested by centrifugation after overnight expression at 22 °C and 250 rpm.

The pellet was resuspended in equilibration buffer (50 mM Tris, 150 mM NaCl, pH 8.2), and cells were destroyed by sonication. After removal of cellular debris by centrifugation (4 °C, 20,000 rpm, 20 min), supernatant was filtered using a 0.45 µM syringe filter and applied onto a Ni-NTA column. The column was washed with 5 column volumes (CV) equilibration buffer and 5 CV wash buffer (50 mM Tris, 150 mM NaCl, 10 mM imidazole, pH 8.2). His tagged mScn-GB1 was eluted with 7 CV elution buffer (50 mM Tris, 150 mM NaCl, 200 mM imidazole, pH 8.2). Elution fractions were evaluated by SDS-PAGE, and the fractions containing the protein of interest were combined, concentrated, and buffer exchanged into equilibration buffer. His-tagged GB1 was cleaved from mScn using Human Rhinovirus 3 C protease (H3C) at a 20:1 GB1-mScn:H3C molar ratio for 2 h at room temperature. The cleavage reaction mixture was filtered using a 0.45 µM syringe filter and loaded onto a Ni-NTA column. The column was washed and eluted as described above. The flowthrough contained mScn, the elution fraction contained His-tagged GB1 and uncleaved fusion protein. The flowthrough and wash fractions were analysed by SDS-PAGE, the ones containing mScn were combined, concentrated, and further purified using size exclusion chromatography over HighLoad 16/600 Superdex 75 pg in sodium phosphate buffer pH 6.0. The purified mScn was collected and concentrated to 15 mg/mL.

### NMR spectroscopy

The NMR sample consisted of 200 µL, 500 µM mSc in 100 mM sodium phosphate pH 6.0 with the addition of 5% D_2_O and DSS (final concentration 1 mM) in a 3 mm NMR tube (Voehler et al. 2006). The following NMR spectra were obtained with a Bruker Avance AV-III 800, equipped with a CPTCI probe, at 298 K and 1 atm: 1H-15 N HSQC, HNCO, HNCA, HN(CA)CO, HN(CO)CA, HNCACB, CBCA(CO)NH (Sattler 1999). Standard Bruker pulse sequences were used and non-linear sampling between 30 and 50% was applied to all 3D acquisitions (Table S1). Data acquisition and processing was performed in Topspin 3.6.4 (Bruker Biospin). ^1^H chemical shifts were referenced to DSS. ^15^N and ^13^C chemical shifts were referenced indirectly based on the ^1^H chemical shifts and the gyromagnetic ratios. For NMR data analysis, CcpNMR AnalysisAssign 3.3.1 was used (Skinner et al. 2016).

### Extent of assignments and data deposition

**Fig. 1:**
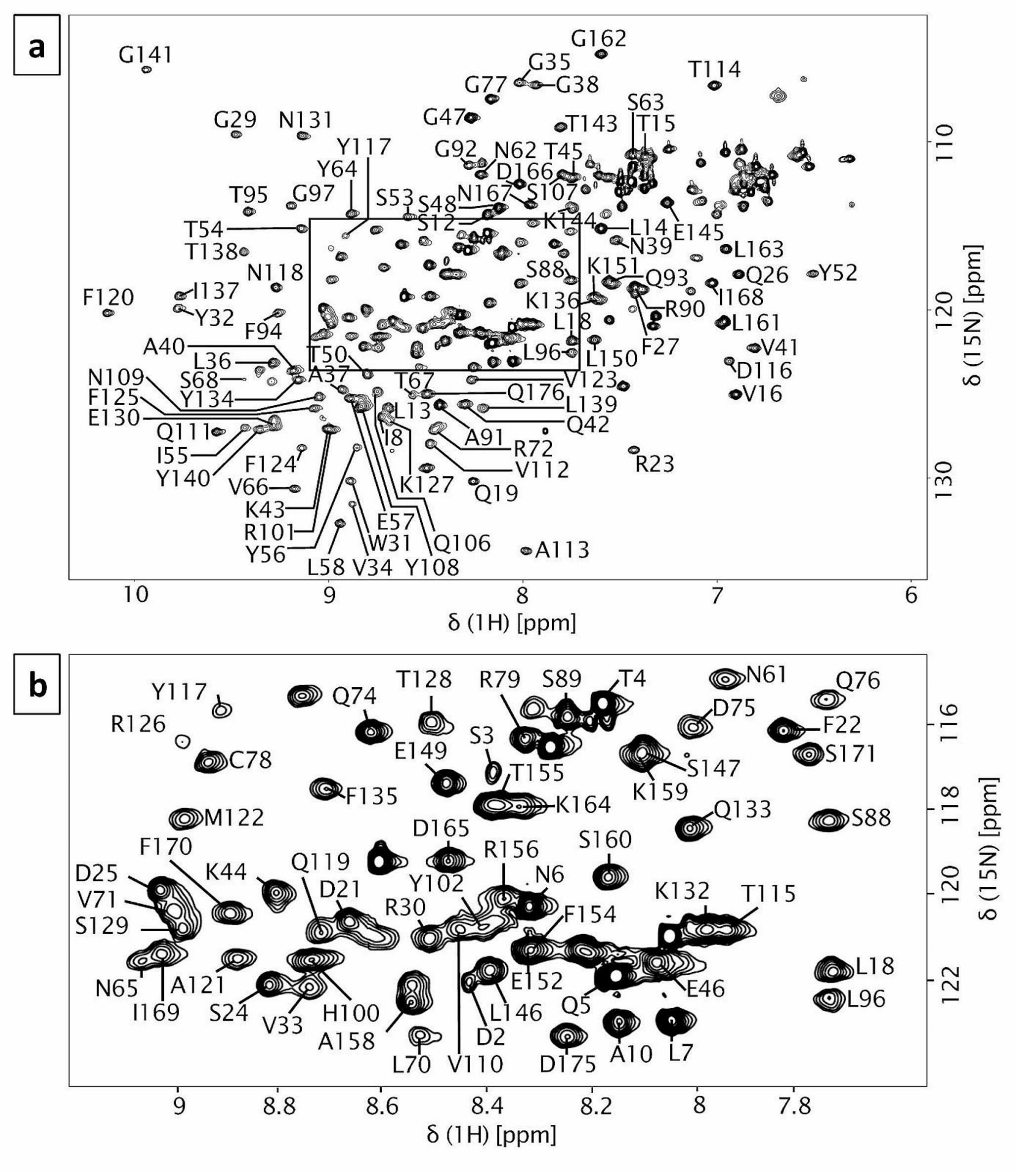
2D ^1^H ^15^N HSQC spectrum annotated with assigned peaks in one letter amino acid code and sequence number. The spectrum was obtained at 800 MHz in 100 mM sodium phosphate buffer pH 6.0. a – HSQC spectrum of mouse siderocalin, b – detail of crowded area indicated in spectrum A

The NMR backbone assignment was based on 3D heteronuclear NMR experiments that were performed on uniformly ^15^N/^13^C labelled mScn. The HSQC spectrum displayed well-dispersed peaks, suggesting mScn was well folded and stable under the chosen conditions. In total, the ^1^H and ^15^N chemical shifts could be identified and successfully assigned for 150 out of 172 assignable residues (87.2%) (Fig. 1). Among the not assigned residues is β-strand D (Y80-V86), as well as Q59, I69, N98, M99, Q104, V105, R142, F157, V172, T174 and the C- and N-terminus with C177-N180 and Q1. The assigned resonances were 86.6% for ^13^Cα (149/172) and 81% for ^13^Cβ (132/163).

Based on the backbone chemical shifts, the secondary structure propensities were predicted using two methods: TALOS-N and the CSI 3.0 web server. TALOS-N predicts backbone torsion angles using a combination of an artificial neural network and a chemical shifts database search for protein motifs with similar properties as the query protein. Where there is no information on chemical shifts, the prediction is based on the amino acid sequence alone (Shen and Bax 2013). The CSI 3.0 web server calculates each residue’s chemical shift index (CSI) – the deviation of the observed chemical shifts from chemical shifts in a random coil – and combines the obtained CSIs with the prediction of torsion angles (TALOS-N), backbone flexibility (random coil index), and fractional accessible surface area (Hafsa et al. 2015). In the case of missing chemical shift information, a curated sequence-chemical shift database is used for the CSI determination and sequence data for TALOS-N predictions (Hafsa and Wishart 2014; Shen and Bax 2013).

**Fig. 2:**
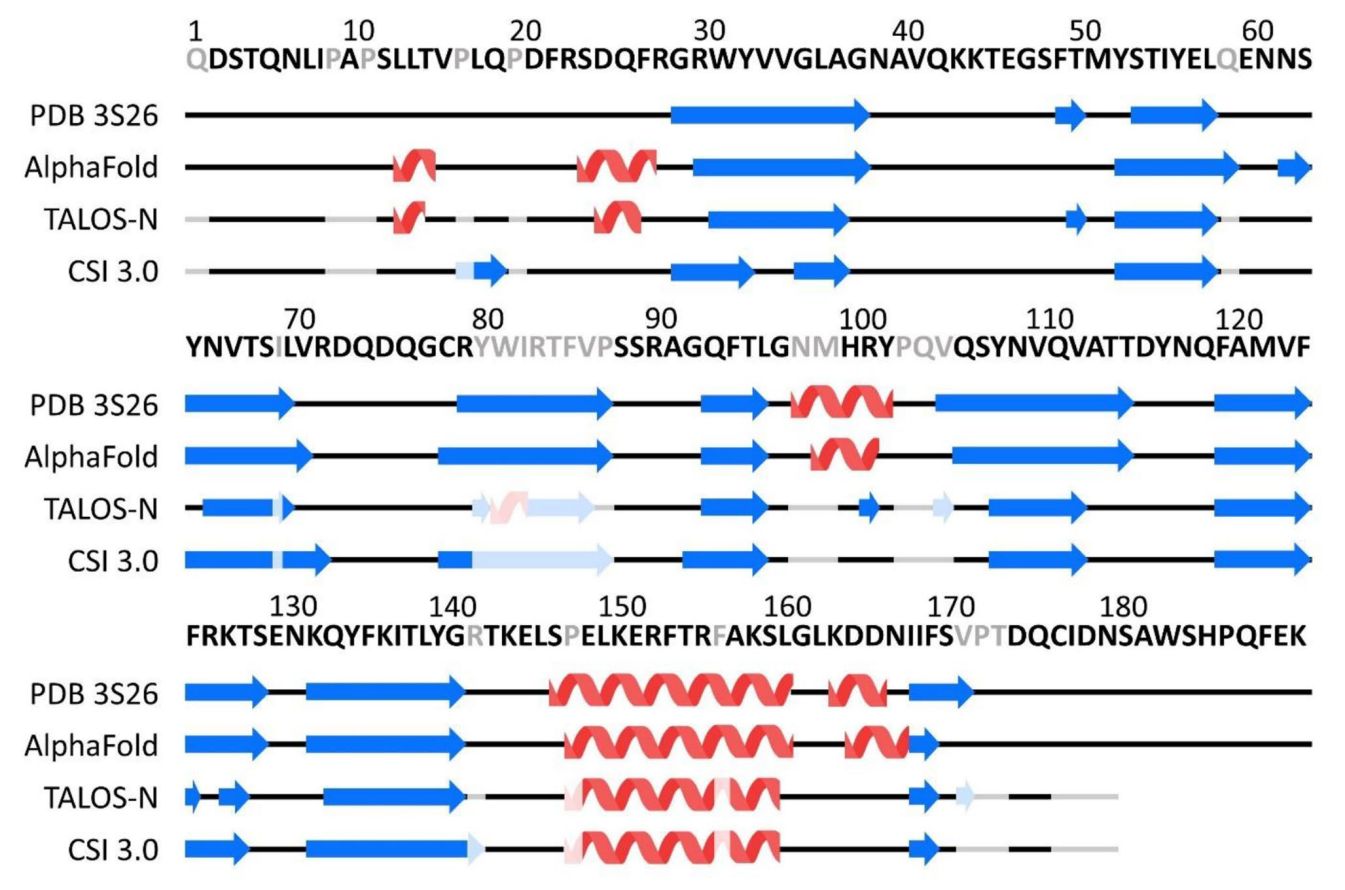
Comparison of the secondary structure elements observed in the mScn crystal structure (3S26) and AlphaFold model with TALOS-N and CSI 3.0 webserver predictions. Residues with missing chemical shift assignments are indicated in grey in the sequence. Secondary structure prediction based solely on the sequence is indicated in lighter colours than the prediction based on chemical shifts

The predictions based on the experimental data were consistent with the crystal structure of mScn (PDB ID 3S26, Bandaranayake et al. 2011) and the model generated by AlphaFold (Jumper et al. 2021), showing the conserved β strands and an α helix typical for the lipocalin protein family (Fig. 2). The TALOS-N predictions showed regions, where the secondary structure differs from the observations in the crystal structure and AlphaFold prediction. These are a short helix at positions 81 and 82 and two very short regions of predicted β strands at positions 101 and 105. The TALOS-N confidence score at these positions is low and, in the case of the α helix, the prediction is solely based on the sequence since chemical shift information is lacking. Based on this, the prediction at these positions can be regarded as low probability. In the CSI 3.0 prediction, the secondary structure of these regions is predicted in concordance with the crystal structure and AlphaFold model.

The observed chemical shifts were compared to the assigned backbone chemical shifts of rat and human siderocalins (81 and 60% sequence identity, respectively) (Åkerström 2006; Coles et al. 1999; Zhang et al. 2009). As expected, the comparison showed good correspondence for homologous regions. We were unable to assign the chemical shifts for one mScn β strand (D4, Y80 – V86) that was successfully assigned in both rat and human siderocalins. For human Scn, increased flexibility has been described for that β strand as well as loop regions (Coles et al. 1999). That kind of flexibility is associated with broadened peaks and low intensities and could be the reason for our missing chemical shift assignments, since they are predominantly located in these regions of high flexibility. The dissimilarities regarding the extent of assignment between mouse, rat and human siderocalin might have been caused by the differences in the used buffer composition. A detailed comparison of the chemical shifts is given in the Supplementary Information.

The ^1^H, ^15^N and ^13^C chemical shift assignments are deposited in the Biological Magnetic Resonance Data Bank (BMRB, http://bmrb.io) under the accession number 52,144.

### Electronic supplementary material

Below is the link to the electronic supplementary material.

Supplementary Material 1

## Data Availability

Chemical shift assignments have been deposited to the Biological Magnetic Resonance Data Bank (BMRB, http://bmrb.io), accession number 52144. Relevant experimental acquisition conditions are summarized in the Supplemental Material.
