# Supplementary material for: Backbone chemical shift and secondary structure assignments for mouse siderocalin
Source: Biomol NMR Assign. 2024 Apr 2;18(1):79–84. doi: 10.1007/s12104-024-10171-9 (PMC11081974; doi:10.1007/s12104-024-10171-9)
Supplement: Supplementary file 1 — Supplementary Material 1 [file 12104_2024_10171_MOESM1_ESM.docx]

**Supplemental Material**

| **Supplemental Table 1: Experimental conditions**. The table shows all relevant acquisition conditions for the experiments reported. In addition to the parameters listed, the transmitters for the amide detect experiments were set to 4.7 ppm for proton, 119.5 ppm for nitrogen and 39, 40, 53, and 172.5 ppm for C_ali_, C_α_C_β_, C_α_, C’, respectively.  **800 MHz** | | | | | | | |
| --- | --- | --- | --- | --- | --- | --- | --- |
| **Sample in 3mm tube** | | | | | | | |
| **Experiment** | **Complex acquisition matrix** | **Sweep width**  **2D: F2 x F1**  **3D: F3 x F2 x F1** | **Number of scans** | **NUS amount** | **spin-lock field** | **Experiment time** |  |
| HSQC | 2k x 512 | 9615 x 2918 Hz | NS=4 |  |  | 0.8 h |  |
| HNCO | 2k x 80 x 128 | 9578 x 2618 x 2554 Hz | NS=8 | 40% |  | 11.2 h |  |
| HNCA | 2k x 80 x 128 | 9578 x 2618 x 6435 Hz | NS=16 | 40% |  | 22.1 h |  |
| CBCAcoNH | 2k x 80 x 128 | 9578 x 2618 x 14077 Hz | NS=16 | 40% |  | 22.8 h |  |
| HNCACB | 2k x 64 x 128 | 9578 x 2618 x 12066 Hz | NS=64 | 50% |  | 111.7 h |  |
| HNcaCO | 2k x 64 x 128 | 9578 x 2618 x 2554 Hz | NS=16 | 30% |  | 12.6 h |  |
| HNCAco | 2k x 64 x 128 | 9578 x 2618 x 6435 Hz | NS=16 | 50% |  | 28.1 h |  |

**Supplemental Table 2: Chemical shift comparison for mouse, rat and human siderocalin.** Positions of differing sequences between the orthologs are indicated in bold.

| **Mouse siderocalin** | | | **Rat siderocalin** | | | **Human siderocalin** | | |
| --- | --- | --- | --- | --- | --- | --- | --- | --- |
| **pos** | **HN** | **N** | **pos** | **HN** | **N** | **pos** | **HN** | **N** |
| Q1 |  |  | Q3 | 8.683 | 121.863 | Q2 |  |  |
| D2 | 8.44 | 122.023 | D4 | 8.238 | 121.191 | D3 | 8.58 | 122.81 |
| S3 | 8.394 | 116.997 | S5 | 8.369 | 116.671 | S4 | 8.51 | 116.81 |
| T4 | 8.183 | 115.355 | T6 | 8.27 | 115.529 | T5 | 8.38 | 115.32 |
| Q5 | 8.157 | 121.893 | Q7 | 8.238 | 122.035 | **S6** | 8.26 | 117.63 |
| N6 | 8.324 | 120.221 | N8 | 8.391 | 120.432 | **D7** | 8.32 | 122.5 |
| L7 | 8.051 | 122.966 | L9 | 8.191 | 123.336 | L8 | 8.06 | 122.64 |
| I8 | 8.725 | 126.288 | I10 | 8.8 | 126.755 | I9 | 8.85 | 128.27 |
| P9 |  |  | P11 |  |  | P10 |  |  |
| A10 | 8.152 | 122.994 | A12 | 8.084 | 123.075 | A11 | 8.5 | 121.75 |
| P11 |  |  | P13 |  |  | P12 |  |  |
| **S12** | 8.18 | 114.259 | P14 |  |  | P13 |  |  |
| L13 | 8.695 | 125.79 | L15 | 8.474 | 127.695 | L14 | 8.52 | 122.64 |
| **L14** | 7.596 | 115.096 | **I16** | 7.902 | 113.179 | **S15** | 7.87 | 112.28 |
| **T15** | 7.37 | 110.814 | **S17** | 7.841 | 117.243 | **K16** | 7.6 | 120.22 |
| V16 | 6.9 | 124.96 | V18 | 7.42 | 127.217 | V17 | 7.4 | 120.27 |
| P17 |  |  | P19 |  |  | P18 |  |  |
| L18 | 7.743 | 121.795 | L20 | 7.818 | 122.452 | L19 | 7.4 | 116.94 |
| Q19 | 8.255 | 130.126 | Q21 | 8.443 | 129.609 | Q20 | 8.52 | 127.49 |
| P20 |  |  | P22 |  |  | **Q21** | 8.94 | 130.35 |
| **D21** | 8.669 | 120.587 | **G23** | 8.646 | 111.985 | **N22** | 9.23 | 120.6 |
| F22 | 7.838 | 116.014 | F24 | 7.602 | 116.519 | F23 | 8.0 | 117.83 |
| **R23** | 7.427 | 128.265 | **W25** | 7.821 | 128.678 | **Q24** | 7.56 | 128.24 |
| **S24** | 8.822 | 122.099 | **T26** | 8.514 | 120.849 | **D25** | 8.7 | 126.45 |
| **D25** | 9.032 | 119.839 | **E27** | 9.667 | 120.632 | **N26** | 9.19 | 121.22 |
| Q26 | 6.887 | 117.831 | **R28** | 6.681 | 115.238 | Q27 | 7.03 | 117.56 |
| F27 | 7.421 | 118.969 | F29 | 7.525 | 119.768 | F28 | 7.52 | 119.51 |
| **R28** | 6.579 | 110.755 | Q30 | 6.695 | 110.167 | Q29 | 6.73 | 109.75 |
| G29 | 9.483 | 109.495 | G31 | 9.532 | 109.275 | G30 | 9.58 | 109.71 |
| R30 | 8.516 | 121.017 | R32 | 8.62 | 121.082 | **K31** | 8.58 | 120.6 |
| W31 | 8.89 | 130.109 | W33 | 8.942 | 130.259 | W32 | 8.99 | 130.53 |
| Y32 | 9.779 | 119.846 | **F34** | 9.92 | 119.998 | Y33 | 9.95 | 120.8 |
| V33 | 8.745 | 122.163 | V35 | 8.791 | 122.201 | V34 | 8.44 | 123.12 |
| V34 | 8.877 | 131.458 | V36 | 9.08 | 132.122 | V35 | 8.45 | 106.74 |
| G35 | 8.018 | 106.423 | G37 | 8.143 | 106.724 | G36 | 8.26 | 106.74 |
| L36 | 9.287 | 123.072 | L38 | 9.333 | 122.882 | L37 | 9.28 | 125.14 |
| A37 | 8.934 | 124.654 | A39 | 9.077 | 125.099 | A38 | 9.14 | 125.45 |
| G38 | 7.932 | 106.578 | G40 | 8.101 | 106.794 | G39 | 7.96 | 105.61 |
| N39 | 7.519 | 115.784 | N41 | 7.717 | 116.912 | N40 | 7.71 | 117.16 |
| A40 | 9.18 | 123.529 | A42 | 9.24 | 123.727 | A41 | 9.49 | 124.77 |
| V41 | 6.804 | 122.204 | V43 | 7.008 | 122.711 | **I42** | 6.76 | 121.31 |
| Q42 | 8.3 | 125.534 | Q44 | 8.135 | 124.901 | **L43** | 7.96 | 124.79 |
| K43 | 8.996 | 127.024 | K45 | 9.18 | 127.806 | **R44** | 8.35 | 123.79 |
| **K44** | 8.808 | 119.948 | E46 | 9.384 | 117.959 | E45 | 8.49 | 126.5 |
| **T45** | 7.748 | 112.073 | **R47** | 7.612 | 116.574 | **D46** | 8.44 | 123.68 |
| **E46** | 8.082 | 121.575 | **Q48** | 7.894 | 117.514 | **K47** | 8.47 | 118.18 |
| **G47** | 8.268 | 108.507 | **S49** | 8.135 | 116.209 | **D48** | 7.91 | 118.69 |
| **S48** | 8.124 | 113.855 | **R50** | 8.443 | 120.66 | **P49** |  |  |
| F49 | 7.994 | 120.788 | F51 | 7.89 | 119.92 | **Q50** |  |  |
| T50 | 8.802 | 123.746 | T52 | 8.83 | 122.317 | **K51** |  |  |
| M51 | 8.435 | 126.827 | M53 | 8.501 | 127.11 | M52 | 8.83 | 123.27 |
| Y52 | 6.505 | 117.775 | Y54 | 6.581 | 118.097 | Y53 | 6.85 | 118.4 |
| S53 | 8.591 | 114.405 | S55 | 8.649 | 114.743 | **A54** | 8.33 | 120.75 |
| T54 | 9.142 | 115.101 | T56 | 9.144 | 115.527 | T55 | 9.34 | 116.71 |
| I55 | 9.433 | 126.926 | I57 | 9.423 | 126.762 | I56 | 9.51 | 127.71 |
| Y56 | 8.855 | 128.125 | Y58 | 8.945 | 128.405 | Y57 | 8.88 | 127.91 |
| E57 | 8.888 | 125.212 | E59 | 8.974 | 124.818 | E58 | 9.13 | 125.98 |
| L58 | 8.943 | 132.614 | L60 | 9.242 | 133.154 | L59 | 8.93 | 130.13 |
| Q59 |  |  | Q61 | 8.984 | 125.21 | **K60** | 9.12 | 127.4 |
| E60 | 8.741 | 121.533 | E62 | 8.907 | 121.303 | E61 | 9.11 | 121.95 |
| **N61** | 7.947 | 114.782 | D63 | 7.817 | 116.5 | D62 | 7.71 | 116.45 |
| N62 | 8.217 | 111.853 | N64 | 8.152 | 111.86 | K63 | 8.02 | 111.2 |
| S63 | 7.433 | 110.739 | S65 | 7.887 | 111.62 | S64 | 7.94 | 111.75 |
| Y64 | 8.884 | 114.214 | Y66 | 9.046 | 114.005 | Y65 | 9.15 | 113.3 |
| N65 | 9.068 | 121.534 | N67 | 9.112 | 121.8 | N66 | 9.37 | 121.15 |
| V66 | 9.178 | 130.551 | V68 | 9.149 | 130.368 | V67 | 9.04 | 130.24 |
| T67 | 8.567 | 124.986 | T69 | 8.722 | 125.838 | T68 | 8.8 | 125.19 |
| S68 | 9.435 | 124.062 | S70 | 9.393 | 124.106 | S69 | 9.43 | 123.88 |
| I69 |  |  | I71 | 8.946 | 125.407 | **V70** | 9.16 | 125.28 |
| L70 | 8.535 | 123.317 | L72 | 8.703 | 124.139 | L71 | 8.54 | 123.88 |
| V71 | 9.008 | 120.327 | V73 | 9.086 | 121.412 | **F72** | 9.27 | 123.01 |
| R72 | 8.458 | 127.079 | R74 | 8.244 | 129.148 | R73 | 8.61 | 127.6 |
| **D73** | 8.225 | 121.183 | **G75** | 9.07 | 117.972 | **K74** | 8.93 | 123.36 |
| **Q74** | 8.628 | 116.042 |  |  |  |  |  |  |
| **D75** | 8.011 | 115.920 |  |  |  |  |  |  |
| Q76 | 7.755 | 115.28 | Q76 | 8.929 | 125.289 | **K75** | 7.99 | 109.33 |
| G77 | 8.165 | 107.401 | G77 | 8.034 | 109.565 | **K76** | 7.59 | 119.4 |
| C78 | 8.941 | 116.758 | C78 | 8.392 | 118.208 | C77 | 8.51 | 117.74 |
| R79 | 8.333 | 116.222 | R79 | 9.436 | 124.271 | **D78** | 9.34 | 127.11 |
| Y80 |  |  | Y80 | 8.553 | 121.709 | Y79 | 8.24 | 118.25 |
| W81 |  |  | W81 | 9.113 | 126.039 | W80 | 9.04 | 124.94 |
| I82 |  |  | I82 | 8.94 | 129.534 | I81 | 9.04 | 130.09 |
| R83 |  |  | R83 | 8.709 | 126.527 | R82 | 8.88 | 126.67 |
| T84 |  |  | T84 | 8.386 | 115.268 | T83 | 8.36 | 114.77 |
| F85 |  |  | F85 | 9.363 | 126.204 | F84 | 9.32 | 126.56 |
| V86 |  |  | V86 | 9.489 | 122.375 | V85 | 9.37 | 121.44 |
| P87 |  |  | P87 |  |  | P86 |  |  |
| S88 | 7.751 | 118.177 | S88 | 7.953 | 118.741 | **G87** | 8.25 | 111.57 |
| S89 | 8.251 | 115.678 | S89 | 8.352 | 116.214 | **C88** | 9.23 | 119.88 |
| R90 | 7.42 | 118.613 | R90 | 7.658 | 119.481 | **Q89** | 7.52 | 116.25 |
| **A91** | 8.43 | 125.577 | P91 |  |  | P90 |  |  |
| G92 | 8.278 | 111.349 | G92 | 8.356 | 113.201 | G91 | 8.33 | 112.73 |
| Q93 | 7.554 | 118.241 | Q93 | 7.725 | 119.679 | **E92** | 7.59 | 118.45 |
| F94 | 9.261 | 120.107 | F94 | 9.543 | 121.696 | F93 | 9.24 | 120.27 |
| T95 | 9.415 | 114.098 | T95 | 9.446 | 114.497 | T94 | 9.3 | 112.81 |
| L96 | 7.749 | 122.441 | L96 | 7.767 | 123.267 | L95 | 7.79 | 122.72 |
| G97 | 9.194 | 113.739 | G97 | 9.435 | 115.259 | G96 | 9.3 | 113.66 |
| N98 |  |  | N98 | 8.928 | 118.607 | N97 | 8.92 | 119.0 |
| **M99** |  |  | I99 | 7.68 | 118.766 | I98 | 7.8 | 119.75 |
| H100 | 8.741 | 121.525 | H100 | 8.471 | 119.007 | **K99** | 8.36 | 118.6 |
| **R101** | 9.006 | 127.026 | S101 | 8.127 | 115.156 | S100 | 8.19 | 114.48 |
| Y102 | 8.412 | 120.737 | Y102 | 7.763 | 121.727 | Y101 | 7.88 | 123.52 |
| P103 |  |  | P103 |  |  | P102 |  |  |
| Q104 |  |  | Q104 | 8.851 | 115.11 | **G103** | 8.96 | 112.28 |
| **V105** |  |  | **I105** | 8.151 | 121.399 | **L104** | 7.92 | 123.61 |
| Q106 | 8.75 | 124.797 | Q106 | 8.942 | 125.349 | **T105** | 8.96 | 116.92 |
| S107 | 7.795 | 111.887 | S107 | 7.898 | 112.288 | S106 | 7.75 | 113.35 |
| Y108 | 8.831 | 125.655 | Y108 | 8.947 | 125.485 | Y107 | 8.85 | 124.72 |
| **N109** | 9.051 | 125.102 | **D109** | 9.032 | 127.128 | **L108** | 9.08 | 132.37 |
| V110 | 8.46 | 120.77 | V110 | 8.44 | 119.669 | V109 | 8.69 | 124.9 |
| Q111 | 9.58 | 127.188 | Q111 | 9.686 | 126.549 | **R110** | 9.5 | 126.05 |
| V112 | 8.479 | 127.884 | V112 | 8.599 | 127.864 | V111 | 8.62 | 127.45 |
| A113 | 7.984 | 134.237 | A113 | 8.055 | 131.302 | **V112** | 8.21 | 132.71 |
| **T114** | 7.012 | 106.606 | **D114** | 6.915 | 112.324 | **S113** | 7.18 | 106.63 |
| T115 | 7.945 | 120.787 | T115 | 8.342 | 121.117 | T114 | 8.47 | 118.29 |
| D116 | 6.936 | 122.983 | D116 | 6.871 | 123.568 | **N115** | 7.07 | 121.46 |
| Y117 | 8.916 | 115.535 | Y117 | 8.911 | 116.08 | Y116 | 9.11 | 115.47 |
| N118 | 9.273 | 118.598 | **D118** | 9.298 | 120.629 | N117 | 9.47 | 118.3 |
| Q119 | 8.724 | 120.844 | Q119 | 8.886 | 120.515 | Q118 | 7.76 | 117.74 |
| F120 | 10.142 | 120.133 | F120 | 10.043 | 120.038 | **H119** | 8.76 | 116.41 |
| A121 | 8.884 | 121.487 | A121 | 8.961 | 121.638 | A120 | 8.94 | 121.53 |
| M122 | 8.987 | 118.138 | M122 | 9.392 | 119.722 | M121 | 9.17 | 120.03 |
| V123 | 8.265 | 124.072 | V123 | 8.421 | 124.496 | V122 | 8.6 | 124.41 |
| F124 | 9.14 | 128.148 | F124 | 9.264 | 128.649 | F123 | 9.09 | 127.85 |
| F125 | 9.076 | 125.779 | F125 | 9.031 | 125.4 | F124 | 9.0 | 124.94 |
| **R126** | 8.988 | 116.251 | **Q126** | 8.877 | 119.656 | **K125** | 8.98 | 120.91 |
| K127 | 8.684 | 126.533 | K127 | 8.959 | 125.239 | K126 | 9.38 | 127.67 |
| T128 | 8.513 | 115.806 | T128 | 8.45 | 121.745 | **V127** | 8.31 | 124.39 |
| S129 | 8.989 | 120.764 | S129 | 9.097 | 121.176 | S128 | 8.93 | 120.31 |
| E130 | 9.283 | 126.818 | E130 | 9.406 | 127.515 | **Q129** | 9.36 | 126.12 |
| N131 | 9.139 | 109.577 | N131 | 9.278 | 109.571 | N130 | 8.85 | 109.42 |
| K132 | 7.984 | 120.786 | K132 | 8.029 | 120.709 | **R131** | 7.92 | 119.93 |
| Q133 | 8.015 | 118.366 | Q133 | 8.069 | 119.655 | **E132** | 8.19 | 120.11 |
| Y134 | 9.16 | 124.09 | Y134 | 9.248 | 125.433 | Y133 | 9.35 | 124.41 |
| F135 | 8.715 | 117.407 | F135 | 8.811 | 118.786 | F134 | 8.9 | 117.71 |
| K136 | 7.61 | 119.346 | K136 | 9.207 | 120.666 | K135 | 9.27 | 122.84 |
| I137 | 9.768 | 119.118 | **V137** | 9.833 | 118.229 | I136 | 9.88 | 122.42 |
| T138 | 9.439 | 116.495 | T138 | 9.521 | 118.122 | T137 | 9.61 | 117.47 |
| L139 | 8.204 | 125.793 | L139 | 8.257 | 125.983 | L138 | 8.5 | 126.78 |
| Y140 | 9.356 | 127.043 | Y140 | 9.632 | 127.504 | Y139 | 9.61 | 127.07 |
| G141 | 9.944 | 105.663 | G141 | 10.051 | 105.785 | G140 | 9.95 | 105.61 |
| R142 |  |  | R142 | 8.788 | 120.963 | R141 | 8.92 | 120.91 |
| T143 | 7.807 | 109.053 | T143 | 7.994 | 109.47 | T142 | 7.98 | 109.04 |
| K144 | 7.741 | 113.923 | K144 | 7.942 | 115.667 | K143 | 7.82 | 114.28 |
| E145 | 7.255 | 113.567 | **G145** | 7.631 | 106.638 | E144 | 7.36 | 113.97 |
| L146 | 8.4 | 121.76 | L146 | 8.135 | 119.842 | L145 | 8.47 | 122.13 |
| S147 | 8.107 | 116.54 | S147 | 8.198 | 116.046 | **T146** | 7.08 | 109.98 |
| **P148** |  |  | **D148** | 8.901 | 121.906 | **S147** | 9.0 | 117.29 |
| E149 | 8.485 | 117.274 | E149 | 8.707 | 119.269 | E148 | 8.8 | 121.15 |
| L150 | 7.628 | 121.71 | L150 | 7.612 | 121.663 | L149 | 7.4 | 120.29 |
| K151 | 7.63 | 119.167 | K151 | 7.951 | 119.699 | K150 | 7.84 | 119.89 |
| E152 | 8.323 | 121.278 | E152 | 8.442 | 118.885 | E151 | 8.74 | 118.82 |
| R153 | 8.207 | 121.351 | R153 | 8.006 | 121.956 | **N152** | 8.24 | 119.2 |
| F154 | 8.316 | 121.272 | F154 | 8.234 | 122.036 | F153 | 8.1 | 122.3 |
| **T155** | 8.385 | 117.795 | **V155** | 8.529 | 121.932 | **I154** | 8.74 | 124.99 |
| R156 | 8.374 | 120.048 | **S156** | 8.449 | 113.778 | R155 | 8.47 | 119.02 |
| F157 |  |  | F157 | 8.39 | 123.935 | F156 | 8.63 | 120.84 |
| A158 | 8.551 | 122.518 | A158 | 8.903 | 123.522 | **S157** | 8.47 | 118.38 |
| K159 | 8.109 | 116.538 | K159 | 8.433 | 116.807 | K158 | 8.56 | 121.39 |
| S160 | 8.174 | 119.536 | S160 | 8.215 | 120.23 | S159 | 8.11 | 119.27 |
| L161 | 6.966 | 120.707 | L161 | 7.112 | 120.547 | L160 | 7.03 | 120.35 |
| G162 | 7.595 | 104.727 | G162 | 7.749 | 106.21 | G161 | 7.76 | 104.68 |
| L163 | 6.951 | 116.319 | L163 | 6.974 | 116.636 | L162 | 7.24 | 118.6 |
| K164 | 8.34 | 117.835 | K164 | 8.408 | 118.108 | **P163** |  |  |
| D165 | 8.48 | 119.123 | D165 | 8.581 | 119.166 | **E164** | 8.91 | 117.72 |
| **D166** | 8.019 | 112.446 | N166 | 8.194 | 111.145 | N165 | 8.32 | 112.63 |
| N167 | 7.963 | 113.674 | N167 | 8.184 | 115.448 | **H166** | 8.23 | 118.13 |
| I168 | 7.023 | 118.352 | I168 | 7.101 | 118.474 | I167 | 7.2 | 118.18 |
| **I169** | 9.029 | 121.371 | V169 | 9.114 | 120.449 | V168 | 9.36 | 123.97 |
| F170 | 8.898 | 120.399 | F170 | 8.98 | 120.533 | F169 | 8.93 | 123.86 |
| S171 | 7.789 | 116.581 | S171 | 7.868 | 116.242 | **P170** |  |  |
| V172 |  |  | V172 | 8.706 | 120.556 | V171 | 8.64 | 122.19 |
| P173 |  |  | P173 |  |  | P172 |  |  |
| T174 |  |  | T174 | 6.628 | 112.55 | **I173** | 6.56 | 113.5 |
| D175 | 8.252 | 123.342 | D175 | 8.357 | 123.499 | D174 | 8.25 | 118.05 |
| Q176 | 8.495 | 124.927 | Q176 | 8.608 | 125.84 | Q175 | 7.11 | 118.71 |
|  |  |  | C177 | 9.086 | 116.629 | C176 | 8.9 | 114.61 |
|  |  |  | I178 |  |  | I177 | 9.0 | 116.8 |
|  |  |  | D179 | 8.365 | 121.567 | D178 | 8.21 | 121.73 |
|  |  |  | **N180** | 7.904 | 118.137 | **G179** | 7.66 | 114.48 |
